# Analysis of Morphologic and Rheological Properties of Hyaluronic Acid Gel Fillers to Body Contouring and Its Clinical Correlation

**DOI:** 10.3390/gels11010065

**Published:** 2025-01-15

**Authors:** Maria Cláudia Almeida Issa, Renata M. M. Viana, Paulo R. de Souza Mendes, Mônica F. Naccache, Priscila R. Varges, Eliana P. Marín Castaño, Eliandre Palermo

**Affiliations:** 1 Department of Internal Medicine (Dermatology), Universidade Federal Fluminense, Niterói 24033-900, RJ, Brazil; 2 Private Practice, São Paulo 04012-080, SP, Brazil; renata27@gmail.com; 3 Rheology Group, Department of Mechanical Engineering, Pontifical Catholic University of Rio de Janeiro, Rio de Janeiro 22451-900, RJ, Brazil; pmendes@puc-rio.br (P.R.d.S.M.); naccache@puc-rio.br (M.F.N.); prvarges@puc-rio.br (P.R.V.); epmarinc@puc-rio.br (E.P.M.C.); 4 Private Practice, São Paulo 05455-000, SP, Brazil; eliandre.palermo@gmail.com

**Keywords:** rheology, hyaluronic acid, dermal filler, body contouring

## Abstract

The demand for minimally invasive body contouring procedures, particularly for gluteal augmentation, has grown significantly. This study evaluates the morphologic and rheological properties of four commercially available hyaluronic acid (HA) fillers used for body contouring and explores their clinical implications. Critical parameters such as storage modulus (G′), loss modulus (G″), complex modulus (G*), and damping factor (tan δ) were measured using oscillatory rheological tests to assess each filler’s elasticity, viscosity, and viscoelastic profile. Scanning Electron Microscopy (SEM) was performed to analyze the microstructure of the fillers, providing insights into their microscopic architecture. The results showed differences in mechanical properties and viscoelastic behavior among the fillers. These variations suggest that the choice of filler may need to be tailored to specific body contouring requirements. Understanding these differences is crucial for achieving the best clinical results and patient satisfaction, helping professionals select the most suitable filler for each case.

## 1. Introduction

Body contouring esthetics surgical and non-surgical procedures are rapidly growing. According to the latest report of the International Society of Aesthetic Plastic Surgery (ISAPS), buttock augmentation shows the most significant increase (56.8%) in surgical procedures, with 820,762 performed worldwide by plastic surgeons in 2022 [1].

Hyaluronic Acid (HA) is a naturally occurring glycosaminoglycan and a vital component of the extracellular matrix (ECM) in all adult animal tissues, particularly abundant in connective tissues such as skin, the eye’s vitreous body, joints, and muscles [2]. In the skin, native, non-crosslinked HA has a short half-life of approximately 12 to 24 h [3]. HA undergoes crosslinking to enhance its longevity and preserve its viscoelastic properties as a dermal filler. This process links HA molecules together, forming a stable, three-dimensional network that resists rapid degradation, thereby allowing HA to remain in the skin for a significantly extended duration. Crosslinked hyaluronic acid fillers are widely utilized for soft tissue volumizing and contouring, offering temporary yet sustained volume over time [2,4,5].

Hyaluronic acid-based (HA) dermal fillers are the most utilized medical device for minimally invasive body contouring, mainly buttock augmentation. Despite recent publications reporting safety and satisfaction with this procedure [6,7,8,9,10,11], multiple factors must be considered on the HA gel such as the type and amount of crosslinker, HA concentration, molecular weight, size of HA particles, manufacturing process, and final formation process of the injectable solution or gel, as these features affect the rheological and physicochemical properties of the HA filler [12]. Different dermal fillers may share the same indications while having different rheological, physical, and chemical features. These properties are directly implicated in the clinical outcome and patient satisfaction. Therefore, understanding them will facilitate the choice of the HA filler for each specific indication, area, and the appropriate anatomic injection plane [13].

The rheological characteristics of hyaluronic acid (HA) fillers can be determined using oscillatory strain amplitude sweep tests and oscillatory frequency sweep tests. Oscillatory strain amplitude sweep tests demonstrate how the viscoelastic properties change with varying amplitudes of deformation. They are crucial for ensuring the gel maintains stability and performance under mechanical stress. Oscillatory frequency sweep tests examine these changes with different frequencies of deformation [14].

Viscoelasticity is a crucial property of HA fillers that exhibits viscous and elastic behaviors when subjected to shear deformation. Four rheological parameters are used to characterize this behavior: the storage modulus (G′), the loss modulus (G″), the tan δ, and the complex modulus (G*) [12,15,16].

- Elastic or storage modulus (G′) assesses the gel’s ability to store energy and return to its original shape, reflecting its elastic characteristics. Higher G′ values indicate a more elastic, firmer material.
- Viscous or loss modulus (G″) measures the energy dissipated due to internal friction during deformation, representing the material’s viscous characteristics.
- The damping Factor (tan δ) indicates the relationship between the material’s viscous and elastic properties, a ratio of G″ to G′; lower tan δ values are associated with higher elasticity.
- Complex modulus (G*) describes the overall viscoelasticity or stiffness of the material, reflecting the filler’s resistance to shape changes.

The complex viscosity (η*) is another important parameter that describes the overall resistance of the HA gel to flow under oscillatory shear [16].

Scanning Electron Microscopy (SEM) is a tool that can be used for evaluating the structure and morphology of hyaluronic acid gels. Its images depict the gel’s microstructure and porosity, which can influence its rheological properties and clinical performance.

While HA fillers have been extensively studied in facial applications, their assessment in the context of body contouring remains underexplored. This study aims to evaluate the morphologic and rheological properties of four commercially available hyaluronic acid (HA) fillers used for body contouring and explores their clinical implications.

## 2. Results and Discussion

The rheological behavior of the selected gels and the morphology are presented below. The analyzed gels are commonly used for gluteal augmentation. The rheometer data showed the values of storage modulus (G′), loss modulus (G″), complex modulus (G*), and tan delta (tan δ) for each filler. The Cryo-SEM images depict the microstructure and morphology of the gels. All rheological tests were performed in triplicate to ensure the accuracy and reproducibility of the results, using different samples.

### 2.1. Rheological Analysis

The results of the rheological tests are presented below. The graphs’ symbols represent the experimental data’s mean value, and the shading illustrates the standard deviation.

**Figure 1:**
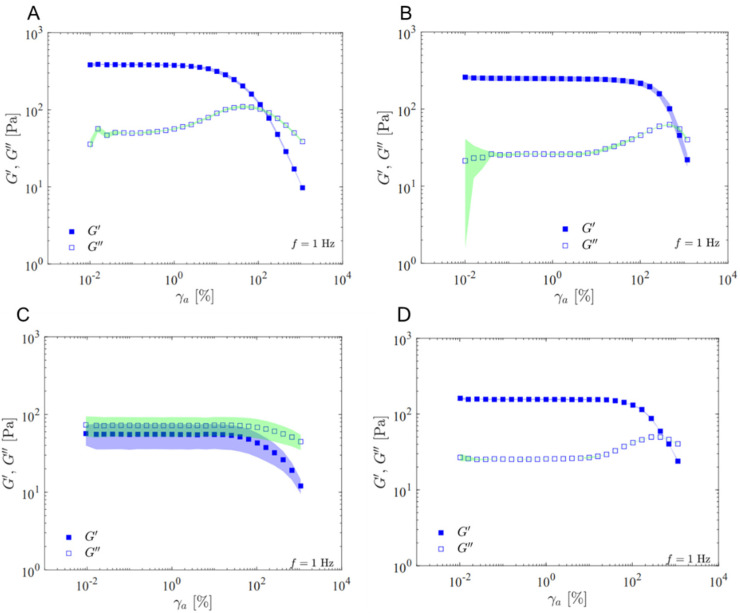
Oscillatory strain amplitude sweep test at 1 Hz, depicting the elastic and viscous moduli as a function of strain amplitude for the following samples: UpM (**A**), SSS (**B**), RBS (**C**), and RSL (**D**).

The oscillatory strain amplitude sweep tests at 1 Hz, detailing the elastic (G′) and viscous (G″) moduli as a function of strain amplitude, are illustrated in Figure 1 for the four samples analyzed. At low amplitude, products UpM, SSS, and RSL present similar curves, showing a G′ greater than G″, indicating a predominance of elastic behavior in the linear viscoelastic region (region where G′ and G″ are constant, γ_a_ ≤ 1%). The sample RBS showed a significant difference in the qualitative profile, with the viscous modulus higher than the storage one, showing a viscous behavior in the linear viscoelastic region (γ_a_ ≤ 10%).

UpM exhibited the highest G′ value (382 Pa), indicating superior support and resilience after deformation. In contrast, RBS showed the lowest G′ value (55 Pa), suggesting a reduced ability to maintain its firmness, volume, and original shape post-application. This may be attributed to the manufacturer’s recommendation for prior dilution with saline solution. RBS showed the highest G″ value (72 Pa), while SSS and RSL exhibited lower G″ values (26 Pa each).

**Figure 2:**
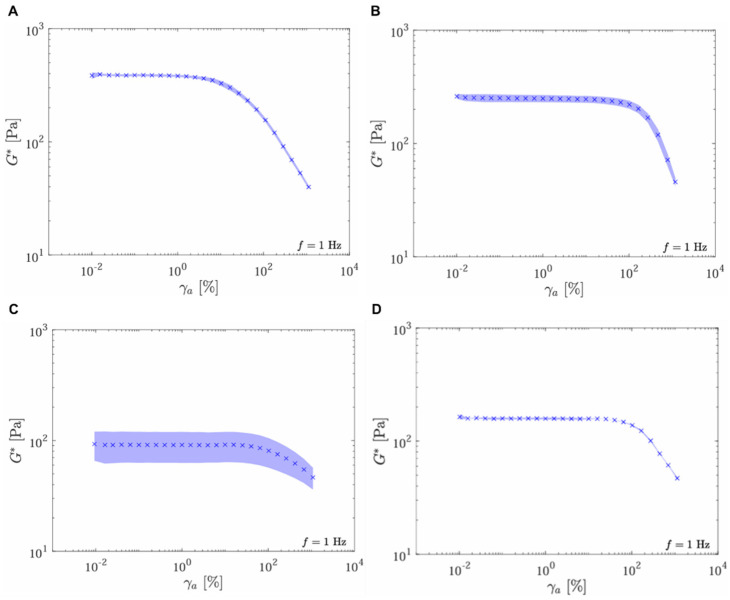
Oscillatory strain amplitude sweep test at 1 Hz. Complex modulus as a function of strain amplitude for the following samples: UpM (**A**), SSS (**B**), RBS (**C**), and RSL (**D**).

Complex modulus (G*) results as a function of strain amplitude were analyzed for the four samples provided. The results are shown in Figure 2, where the x-axis represents the strain (γ_a_ [%]), and the y-axis represents the complex modulus (G* [Pa]). All measurements are conducted at a frequency of 1 Hz. Products UpM (G* 386), SSS (G* 250), and RSL (G* 158) show a high initial G* value that decreases with increasing strain. The complex modulus (G*) offers insights into gels’ overall stiffness and deformation resistance. It measures the total energy required to deform the material under shear stress. It indicates the difficulty in altering the shape of individual crosslinked filler units [16]. UpM and RSL showed the highest G* values (386 Pa and 158 Pa, respectively). In contrast, the RBS (G* = 91 Pa) gel has a different profile with lower initial stiffness. It shows a complex response to strain, indicating it may be less elastic and more prone to deformation under lower strains than the other fillers.

**Figure 3:**
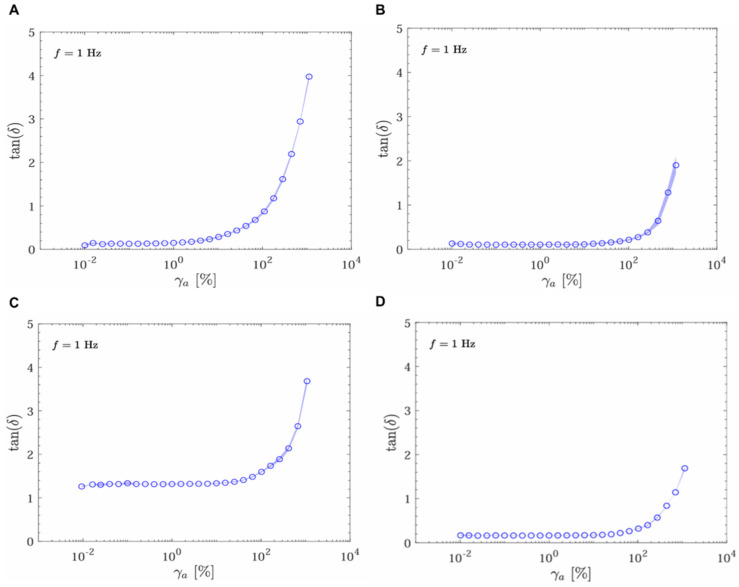
Oscillatory strain amplitude sweep test at 1 Hz. Tangent of the phase angle as a function of strain amplitude for the following samples: UpM (**A**), SSS (**B**), RBS (**C**), and RSL (**D**).

Figure 3 shows the damping factor or loss tangent (tan(δ) of the analyzed products as a function of strain (γ_a_ [%]), at a frequency of 1 Hz. The loss tangent, tan(δ), is the ratio of the loss modulus (G″) to the storage modulus (G′), providing insight into the material’s viscoelastic behavior, measuring whether a filler exhibits more gel-like or liquid-like behavior [16].

Tan(δ) of products UpM, SSS, and RSL remains low, 0.13, 0.10, and 0.17, respectively, and relatively constant at low strains, indicating a predominantly elastic behavior. However, it becomes more viscous at higher strains, indicating a potential for deformation under larger forces. The results show an increase in tan(δ) with increasing strain, marking a transition from elastic to viscous behavior. The strain threshold at which this transition occurs varies among the products. In contrast, sample RBS shows a much higher value tan(δ) value of 1.32, behaving more like a viscous liquid than a gel (tan δ > 1) in all ranges of strain.

**Figure 4:**
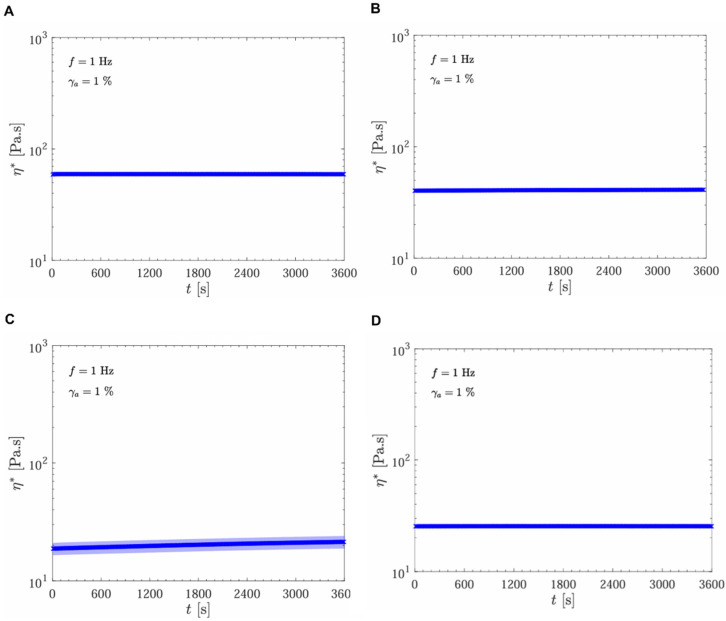
Oscillatory time sweep test at 1 Hz and γ_a_ = 1%: complex viscosity as a function of time for the following samples: UpM (**A**), SSS (**B**), RBS (**C**), and RSL (**D**).

Complex viscosity (η*) is plotted as a function of time under controlled frequency (f = 1 Hz) and strain (γ_a_ = 1%). Across all samples, viscosity remained relatively stable over the 3600 s, with minimal fluctuations in the viscoelastic profile (Figure 4). Gels UpM and SSS, in the top row, displayed consistent viscosity values around 10^2^ Pa·s, indicating a robust structural integrity that remains stable over time. This stability suggests that these gels maintain their mechanical properties under prolonged time of applied stress, which is critical for ensuring predictable behavior when injected into large body areas and in large amounts.

**Figure 5:**
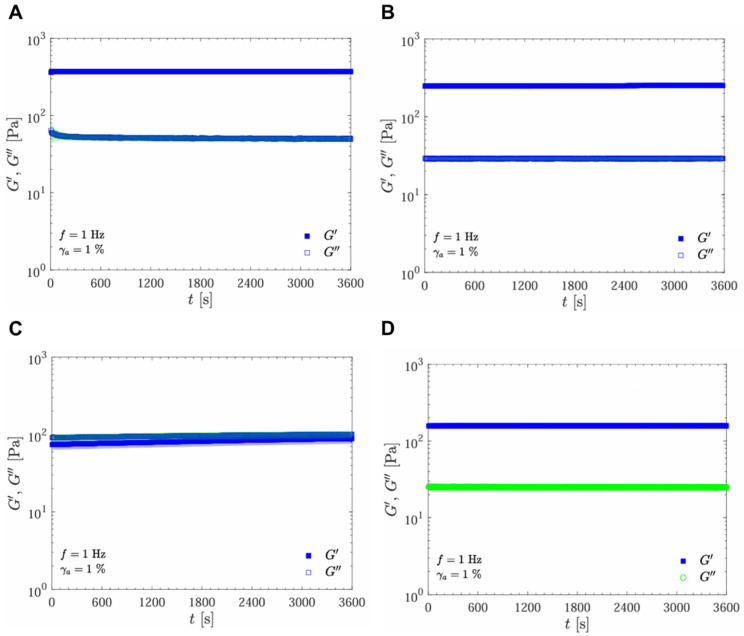
Oscillatory time sweep test at 1 Hz and γ_a_ = 1%: elastic (G′) and viscous (G″) moduli as a function of time for the following samples: UpM (**A**), SSS (**B**), RBS (**C**), and RSL (**D**).

The oscillatory time sweep test results demonstrate different stability levels among the samples (Figure 4 and Figure 5). The UpM and RSL samples exhibited high stability, with G′, G″, and η∗ remaining consistent over time (Figure 4A,D and Figure 5A,D). The SSS sample also showed stable behavior, with elastic effects predominating and constant moduli and complex viscosity throughout the analysis period (Figure 4B and Figure 5B). The RBS sample showed increased elastic and viscous moduli and complex viscosity over time (Figure 4C and Figure 5C).

Despite differences among their results, three HA gels (UpM, SSS, and RSL) presented physical properties that might be suitable for body contouring. UpM stands out with the highest G′ among all samples, indicating the strongest elastic behavior. This suggests that UpM is the most suitable for body contouring applications that require significant lifting and projection such as gluteal augmentation. Its high elasticity ensures that the gel maintains its structure under mechanical stress, providing lasting results. While still elastic with high G′, SSS exhibits lower G′ values than UpM.

RSL displays a significant difference between G′ and G″, with G′ remaining constant around 10^2^ Pa and G″ much lower, indicating inferior rheological properties for body contouring compared to UpM and SSS.

RBS gel exhibits a more balanced relationship between G′ and G″, but its considerably lower G′ compared to the others suggests it may not be adequate for gluteal treatment, likely due to the product’s recommended dilution with saline solution prior to application.

Table 1 summarizes the rheology properties of the analyzed products, detailing the mean values of G′, G″, G*, and tan (δ).

This study offers valuable insights into the rheological behavior of hyaluronic acid (HA) fillers in vitro. However, we hypothesize about their potential behavior in body contouring applications, as there are currently no studies that directly link rheological properties to clinical outcomes in this context. In facial procedures, fillers with higher storage modulus (G′) values are recommended for achieving lifting effects and structural definition [17]. Similarly, for gluteal augmentation, fillers with higher G′ values may be ideal for enhancing the lateral and upper regions of the buttocks, providing both lift and projection. This article presents an initial analysis aimed at characterizing the rheological properties of products used in body contouring, with a particular focus on gluteal augmentation.

### 2.2. Scanning Electron Microscopy Images

**Figure 6:**
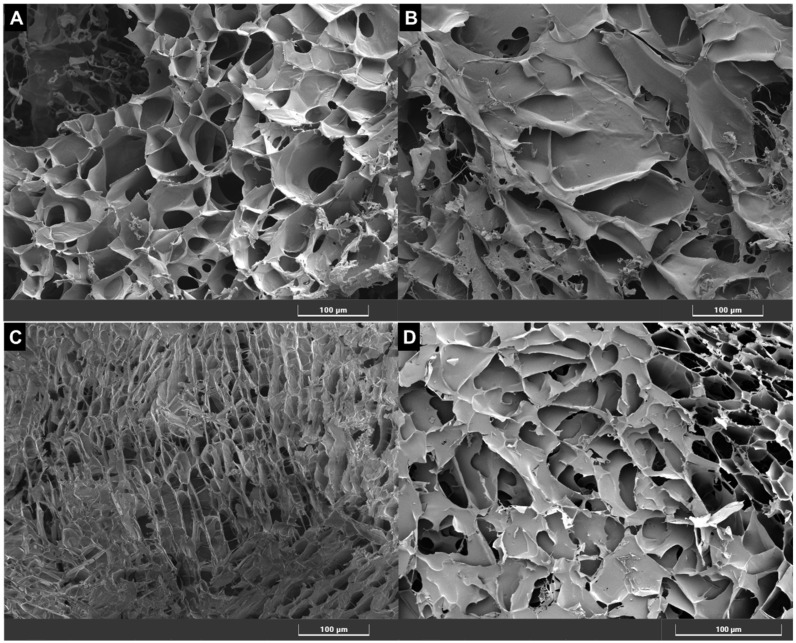
SEM analysis showing the morphological characteristics of hyaluronic acid samples labeled (**A**) UpM, (**B**) SSS (**C**), RBS, and (**D**) RSL.

To provide supplemental structural information in addition to the results of the rheological properties, Scanning Electron Microscopy (SEM) was performed to investigate the differences in the ultrastructure of these materials. The results are shown in Figure 6. The samples were processed using the cryogenic method for sample preparation, and the scale bar in each image represents 100 μm.

The SEM analysis of the fillers reveals distinct micromorphological characteristics and all gels show a porous interconnected network structure. UpM gel displays a highly porous and interconnected structure with significant void spaces, appearing to have a more organized architecture with uniformly distributed pores, which may suggest a well-structured matrix (Figure 6A). In contrast, SSS shows a denser and more compact structure, characterized by smaller pores and a less porous, tightly packed arrangement (Figure 6B). RBS presents a fibrillar structure with intertwined layers, exhibiting a more linear arrangement with fewer visible voids, indicating a dense and aligned configuration, which seems to be consistent with the lower values of the viscous and elastic moduli obtained in the rheological tests (Figure 6C). Finally, RSL reveals a moderately porous structure, with a mix of large and small pores distributed throughout (Figure 6D). The porosity pattern in RSL appears less uniform, with varying pore sizes scattered across the material.

A study by Flynn et al. [18] used Scanning Electron Microscopy (SEM) to examine the microstructure of three HA fillers commonly used for facial procedures, highlighting differences in particle distribution and uniformity, which may influence their clinical applications. Similarly, while our study provides valuable insights into the rheological properties and microstructure of HA fillers used for body contouring, it remains speculative to directly correlate these characteristics with clinical outcomes due to the lack of available literature on this topic. The findings from this study contribute to a deeper understanding of the fillers’ structural attributes, but more data are needed to establish definitive correlation between these properties and their clinical performance.

### 2.3. Application of Hyaluronic Acid Fillers in Gluteal Harmonization and Volumization

HA fillers for gluteal harmonization and volumization have emerged as a popular alternative to traditional surgical procedures owing to their minimally invasive nature [2,4,5]. Among the currently utilized techniques employed for gluteal augmentation—implant-based procedures, autologous fat grafting, local tissue rearrangement or flaps, injection of hyaluronic acid gel or other non-permanent fillers, and silicone injections—HA fillers present distinct advantages. They offer a minimally invasive option with no downtime and a lower risk of complications compared to surgical methods. While implant-based and fat grafting approaches often entail significant downtime and higher complication rates, HA fillers provide a favorable safety profile, immediate results, and the potential for adjustment or removal. This flexibility and their ability to deliver substantial esthetic enhancements without extensive recovery time positions crosslinked HA fillers as a preferred choice for gluteal augmentation [19,20,21,22]. However, one limitation is the high cost, particularly for body areas like the gluteal region, which require large volumes of filler. Additionally, the results are temporary, as the filler degrades over time, necessitating maintenance treatments for a sustained outcome. This temporal nature of the results can be seen as either an advantage or a disadvantage, depending on the patient’s goals. For those seeking a non-permanent solution, it offers flexibility, but for patients desiring long-lasting outcomes, it may be less suitable. Contraindications for HA fillers include active skin infections, autoimmune disorders, and hypersensitivity to hyaluronic acid or any of the product’s components.

Understanding the rheological properties of commercially available fillers is critical for practitioners to select the most appropriate product for each individual case. This study represents the first investigation into the rheological and morphological characteristics of HA fillers currently available in Brazil and most used for body contouring, particularly for gluteal augmentation, addressing a significant gap in the current literature. Most existing research has primarily concentrated on fillers intended for facial regions [15,16], highlighting the need to comprehensively analyze the unique demands and applications associated with body contouring.

### 2.4. Application of Hyaluronic Acid Fillers in Other Body Areas

While gluteal augmentation remains one of the most recognized uses of hyaluronic acid (HA) fillers in body contouring, their application extends to other areas, such as the abdomen, pectoral region, and intimate areas. In the abdomen, HA fillers have gained prominence for their ability to enhance muscle definition and improve esthetic contours, particularly in patients with low body fat. This technique involves precise injection of the filler into specific anatomical planes, such as the subcutaneous fat, to create light and shadow effects that enhance the appearance of abdominal muscles. These principles are similarly applicable to the pectoral region and oblique muscles, where HA fillers can be used to refine contours and achieve satisfactory esthetic outcomes. These approaches employ fillers with large particle sizes and high elastic modulus [23,24].

In penile augmentation, HA is primarily utilized to increase penile girth, providing immediate and satisfactory results for patients. Recent studies indicate that HA is particularly effective in enhancing penile diameter and improving sexual satisfaction post-procedure [25,26,27].

HA injections have proven also to be an effective option for restoring volume and rejuvenating the labia majora, particularly in cases of hypotrophy associated with aging. Beyond enhancing esthetic appearance, this procedure contributes to functional comfort, with positive effects on tissue elasticity and overall well-being [28,29,30]. It is essential to further explore the rheological properties of hyaluronic acid fillers, as their expanding use in body contouring applications demands a deeper understanding to optimize their efficacy and safety.

## 3. Conclusions

The findings from this study provide an in-depth analysis of the rheological and morphological characteristics of hyaluronic acid fillers for body contouring, with a particular focus on gluteal augmentation. These properties offer critical insights into the fillers’ potential performance, enabling practitioners to select products that align with individual patient needs. Notably, the elastic modulus (G′) serves as a key parameter for identifying products that may provide superior lifting and structural integrity, while the damping factor (tan δ) highlights the viscoelastic balance required for predictable behavior under mechanical strain. Among the HA fillers studied, the UpM gel demonstrated the highest G′ and a well-balanced damping factor (tan δ) making it the most promising candidate for applications requiring high support and projection, followed by the SSS gel. Additionally, the inclusion of lidocaine in the formulation of the gel may represent an ancillary benefit, potentially improving patient comfort during the application process.

Furthermore, the results indicate that the gels exhibited excellent stability, as the rheological properties remain consistent over time during the experiments. Future research should focus on translating these findings into clinical practice to establish stronger correlations between microstructure, rheological properties, and clinical outcomes.

## 4. Materials and Methods

### 4.1. Sample Selection/Material

Four hyaluronic acid gel fillers commercially available in the market and commonly used for body contouring were selected: UP Max (UpM), by Ilikia—CG Bio Co., Ltd., Hwaseong-si, Republic of Korea; Rennova Body Shape (RBS)—Allanmar International Company S.R.L, Rosario, Argentina; Rennova Lift Shape Lido (RLS lido)—HUMEDIX Co. Ltd., Jecheon-si, South Korea; and Sofiderm Derm Sub Skin (SSS)—Hangzhou Techderm Biological Products Co., Ltd., Hangzhou, China. The information on these fillers, available in the Instructions for Use (IFU) or on the manufacturer’s website, is presented in Table 2. The products UP Max and Rennova Lift Shape Lido contain 0.3% and 0,32% lidocaine, respectively, in their composition. For the rheological tests, RBS [11] gel was diluted according to the supplier’s instructions, for which 3 mL of filler was diluted in 3 mL of saline solution. All other fillers were analyzed as they were presented in the syringe.

### 4.2. Rheological Testing

Rheological tests were conducted using a TA Instruments Discovery Hybrid Rheometer (DHR-3). Grooved parallel plates with a diameter of 60 mm and a gap of 0.5 mm were selected as the geometry for all tests, employing the same gel volume for each. The selection of the appropriate geometry, considering the material’s specific characteristics, is a crucial stage in the testing process. The HA fillers tested in this study presented the expected gel-like behavior, characteristic of structured materials that present yield stress. The yield stress is the minimum value of stress required to break the fluid microstructure. This material behaves as a viscoelastic solid if the applied stress is below the yield stress. At this level of stress, they present very high viscosity values. Once the yield stress is overcome, it behaves as a purely viscous shear-thinning fluid. A rough or grooved geometry, recommended in the rheometer for the yield stress (structured) materials to mitigate the typical apparent wall slippage observed in structured materials at low strain rates, was performed at a constant temperature, as all the other tests [31,32,33,34]. Temperature control was achieved using the Peltier system throughout the experiments. Each type of analysis was performed in triplicate to ensure repeatability.

The samples’ viscoelastic behavior was assessed through oscillatory strain amplitude sweep tests [35], conducted at a frequency of 1 Hz with a strain amplitude (γ_a_) ranging from 0.01% to 1000%. In this test, a sinusoidal strain is applied, and the resulting stress is measured. The main function of this test is to identify the Linear Viscoelastic Regime (LVR), where the elastic or storage (G′) and viscous or loss (G″) moduli depend on frequency only and remain unaffected by changes in the imposed strain amplitude. By correlating the fluid microstructure with the mechanical linear response, this test provides insights into the material’s behavior [35]. It is worth mentioning that the moduli outside LVR, particularly at higher strain amplitudes, lose their physical significance. Thus, this test defines the maximum value of the strain amplitude γ_a,max_ so that the material structure preserves the same characteristics as in the quiescent state. Other qualitative information that can be obtained in this test is regarding the yield stress of the material, usually taken as the crossover point (when G′ = G″). Although this test does not provide a precise value, it can provide an estimate for the value of the yield stress. The other oscillatory tests, such as the time and frequency sweep tests, were conducted exclusively within the linear viscoelastic regime.

The oscillatory time sweep test measured the storage and loss moduli over time and monitored changes in the microstructure, including issues such as sample evaporation, sedimentation, and thixotropy effects [35]. This test involves applying a constant frequency and strain amplitude within the LVR while subjecting the material to a sinusoidally varying strain over time. The behavior of the elastic and viscous moduli is then observed over time. Samples with stable microstructures exhibit constant and time-independent moduli.

The oscillatory frequency sweep test is a valuable tool for evaluating the mechanical response of the material’s microstructure in its quiescent state. In this test, a constant strain amplitude is imposed within the LVR, while the storage and loss moduli behavior are observed with varying frequencies [35].

The measurements obtained in the oscillatory tests included the storage modulus (G′), loss modulus (G″), complex modulus (G*), and damping factor (tan δ) [2]. The equations for these parameters are:

$$G*=\sqrt{G^{\prime 2}+{G\prime \prime }^{2}},$$

η* = G*/ω where ω is the angular frequency.

Tan (δ) = G″/G′, where δ is the phase angle.

#### Comparative Analysis

The products were compared based on their rheological parameters to determine their suitability for clinical applications. Specifically, the storage modulus (G′) was used to evaluate each gel’s firmness and structural support, while the damping factor provided insight into its energy dissipation behavior.

Pairwise comparisons were made between products to assess significant differences in their rheological behavior.

### 4.3. Scanning Electron Microscopy (SEM) Evaluation

Scanning Electron Microscopy (SEM) was employed to observe hyaluronic acid samples using the Tescan Clara scanning electron microscope (TESCAN, Brno, Czech Republic). Cryogenic sample preparation (Quorum) was utilized for analysis. Cryo-SEM is a technique that allows the visualization of a liquid sample microstructure, which involves freezing the sample and removing water via sublimation. However, it is important to notice that the sample preparation process must be carefully performed to avoid damage to the original microstructure.

For the cryogenic preparation method, samples were fast frozen in liquid nitrogen at −210 °C under vacuum. The sample surfaces were then trimmed with a blade to remove the excess material and to achieve a flat surface for better focus. Sublimation was conducted for 5 min at −60 °C to remove water. Subsequently, samples were sputter-coated with platinum using the Quorum cryogenic system. SEM analysis was performed at 5 kV voltage and 164 pA current, with a working distance of 10 mm. Images were obtained at a magnification of 500×.

These SEM images provide detailed insights into the fluid microstructure and morphology of the hyaluronic acid samples prepared using the cryogenic method. The results can be qualitatively compared to fluid rheology, in an attempt to explain the material behavior.

### 4.4. Statistical Evaluation

The results for G′, G″, and G* were expressed as mean values for each product. Standard deviations were calculated to assess the variability of the measurements over time. A time-based stability analysis was conducted to evaluate whether the rheological properties of each gel remained constant throughout the testing period. This was performed to ensure the gels maintain their structural integrity over time.

The damping factor (tan δ = G″/G′) was calculated to differentiate between elastic and viscous behavior, helping to classify the gels as predominantly elastic (tan δ < 1) or viscous (tan δ > 1).

## Figures and Tables

**Table 1:** Mean values of viscoelastic properties for each investigated product.

Rheology	UpM	SSS	RBS	RSL
ELASTIC MODULUS (G′) [PA]	382	248	55	156
VISCOUS MODULUS (G′′) [PA]	50	26	72	26
COMPLEX MODULUS (G*) [PA]	386	250	91	158
DAMPING FACTOR (TAN Δ)	0.13	0.10	1.32	0.17

**Table 2:** Characteristics of hyaluronic acid gels provided by the manufacturers.

Product	AH Concentration	Cross-Linking Agent	Cross-Linking Technology
UpM	20 mg/mL	BDDE	R2
SSS	20 mg ± 3 mg/mL	BDDE	MCLPE
RBS	30 mg/mL	BDDE	Matrix XXDens
RSL	23 mg/mL	BDDE	HI Molecular

## Data Availability

All generated data were presented at this publication.

## References

[1] The International Society of Aesthetic Plastic Surgery (ISAPS) (2023). ISAPS Internacional Survey on Aesthetic/Cosmetic Procedures Performed in 2022.

[2] Brandt F.S., Cazzaniga A. (2008). Hyaluronic acid gel fillers in the management of facial aging. Clin. Interv. Aging.

[3] Succi I.B., da Silva R.T., Orofino-Costa R. (2012). Rejuvenation of Periorbital Area: Treatment with an Injectable Nonanimal Non-Crosslinked Glycerol Added Hyaluronic Acid Preparation. Dermatol. Surg..

[4] Hong G.-W., Wan J., Park Y., Yoo J., Cartier H., Garson S., Haykal D., Yi K.-H. (2024). Manufacturing Process of Hyaluronic Acid Dermal Fillers. Polymers.

[5] Greene J.J., Sidle D.M. (2015). The Hyaluronic Acid Fillers. Facial Plast. Surg. Clin. N. Am..

[6] Mortada H., Alkadi D., Saqr H., Sultan F., Alturaiki B., Alrobaiea S., Aljaaly H.A., Arab K., Arkoubi A.Y. (2023). Effectiveness and Role of Using Hyaluronic Acid Injections for Gluteal Augmentation: A Comprehensive Systematic Review of Techniques and Outcomes.

[7] de Matos Lourenço L., Colla L.A., de Noronha M.G.O., Izzo T.R., Sigrist R. (2024). Square technique—A treatment for cellulite with large size particle hyaluronic acid. Ski. Health Dis..

[8] Lourenço L.M., de Noronha M.G.O., Colla L.A., Izzo T.R., Sigrist R., Braz A. (2022). LL body contour technique—A new way of gluteal contouring and augmentation with hyaluronic acid filler. J. Cosmet. Dermatol..

[9] Santorelli A., Cerullo F., Salti G., Avvedimento S. (2023). Gluteal Augmentation with Hyaluronic Acid Filler: A Retrospective Analysis Using the BODY-Q Scale. Aesthetic Plast. Surg..

[10] Arantes M. (2024). A Non-Surgical Approach to Treat Gluteal Asymmetry Due to Large Hemangioma: A Case Report. JOJ Dermatol. Cosmet..

[11] Faria G., Boggio R., Bellini M. (2023). Glutaeal remodelling protocol: Volumization with hyaluronic acid and collagen biostimulation with poly-L-lactic acid. Ski. Health Dis..

[12] Pierre S., Liew S., Bernardin A. (2015). Basics of dermal filler rheology. Dermatol. Surg..

[13] Sundaram H., Voigts B., Beer K., Meland M. (2010). Comparison of the rheological properties of viscosity and elasticity in two categories of soft tissue fillers: Calcium hydroxylapatite and hyaluronic acid. Dermatol. Surg..

[14] Byron B.R., Armstrong R.C., Hassager O. (1977). Dynamics of Polymer Liquids Vol. 1 Bird Armstrong Curtis Hassager.

[15] Fagien S., Bertucci V., Von Grote E., Mashburn J.H. (2019). Rheologic and Physicochemical Properties Used to Differentiate Injectable Hyaluronic Acid Filler Products. Plast. Reconstr. Surg..

[16] Fundarò S.P., Salti G., Malgapo D.M.H., Innocenti S. (2022). The Rheology and Physicochemical Characteristics of Hyaluronic Acid Fillers: Their Clinical Implications. Int. J. Mol. Sci..

[17] Perera G.G.G., Argenta D.F., Caon T. (2024). The rheology of injectable hyaluronic acid hydrogels used as facial fillers: A review. Int. J. Biol. Macromol..

[18] Flynn T.C., Thompson D.H., Hyun S.-H., Howell D.J. (2015). Ultrastructural Analysis of 3 Hyaluronic Acid Soft-Tissue Fillers Using Scanning Electron Microscopy. Dermatol. Surg..

[19] Oranges C.M., Haug M., Schaefer D.J. (2016). Body Contouring. Plast. Reconstr. Surg..

[20] Hedén P., Sellman G., von Wachenfeldt M., Olenius M., Fagrell D. (2009). Body Shaping and Volume Restoration: The Role of Hyaluronic Acid. Aesthetic Plast. Surg..

[21] Shah B. (2018). Complications in Gluteal Augmentation. Clin. Plast. Surg..

[22] Crabai P., Marchetti F., Santacatterina F., Fontenete S., Galera T. (2024). Nonsurgical Gluteal Volume Correction with Hyaluronic Acid: A Retrospective Study to Assess Long-term Safety and Efficacy. Plast. Reconstr. Surg. Glob. Open.

[23] Dziabas D., Kasai M., HD R.V.C.F. (2024). A Minimally Invasive Technique for Male Body Contouring: A Pilot Study. Plast. Reconstr. Surg. Glob. Open.

[24] Lourenço L.M., Di Sessa D., Tebet A.C.F., de Noronha M.G.O., de Medeiros H.L., Sigrist R. (2024). Hyaluronic High Definition Fill Technique. J. Cosmet. Dermatol..

[25] Kim D.W., Jeong H.C., Ko K., Yang D.Y., Kim J.K., Lee S.H., Kim T.H., Lee W.K. (2024). Which Dermal Filler is Better for Penile Augmentation for Aesthetic Purposes? A Prospective, Single-Surgeon Study Based on Real-World Experience. World J. Mens. Health.

[26] Kusumaputra A., Setiawan M.R., Soebadi M.A., Wirjopranoto S. (2023). Efficacy and complications of hyaluronic acid and polylactic acid for penile augmentation: A systematic review and meta-analysis. Ann. Med. Surg..

[27] Boiko M.I., Notsek M.S., Boiko O.M. (2023). The Efficacy of Injection Penile Girth Enhancement as an Option for Small Penis Syndrome Management. Aesthet. Surg. J..

[28] Tarabini F., Rozemberg L., Zapata-Sudo G., Braz A. (2023). A Novel Hyaluronic Acid Filling Technique for Restoring Volume of the Labia Majora. Cureus.

[29] Hexsel D., Dal’Forno T., Caspary P., Hexsel C.L. (2016). Soft-Tissue Augmentation with Hyaluronic Acid Filler for Labia Majora and Mons Pubis. Dermatol. Surg..

[30] Fasola E., Gazzola R. (2016). Labia Majora Augmentation with Hyaluronic Acid Filler: Technique and Results. Aesthet. Surg. J..

[31] Varges P.R., Costa C.M., Fonseca B.S., Naccache M.F., De Souza Mendes P.R. (2019). Rheological Characterization of Carbopol^®^ Dispersions in Water and in Water/Glycerol Solutions. Fluids.

[32] Buscall R., McGowan J.I., Morton-Jones A.J. (1993). The rheology of concentrated dispersions of weakly attracting colloidal particles with and without wall slip. J. Rheol..

[33] Barnes H.A. (1995). A review of the slip (wall depletion) of polymer solutions, emulsions and particle suspensions in viscometers: Its cause, character, and cure. J. Nonnewton Fluid. Mech..

[34] Barnes H.A. (2000). Measuring the viscosity of large-particle (and flocculated) suspensions—A note on the necessary gap size of rotational viscometers. J. Nonnewton Fluid. Mech..

[35] Lorenc Z.P., Öhrlund Å., Edsman K. (2017). Factors Affecting the Rheological Measurement of Hyaluronic Acid Gel Fillers. J. Drugs Dermatol..

